# Ecological momentary assessment of mind-wandering: meta-analysis and systematic review

**DOI:** 10.1038/s41598-023-29854-9

**Published:** 2023-02-18

**Authors:** Issaku Kawashima, Tomoko Hinuma, Saori C. Tanaka

**Affiliations:** 1 Brain Information Communication Research Laboratory Group, Advanced Telecommunications Research Institute International (ATR), Kyoto, Japan; 2 Division of Information Science, Graduate School of Science and Technology, Nara Institute of Science and Technology, Nara, Japan

**Keywords:** Psychology, Human behaviour

## Abstract

Mind-wandering (MW) is a universal human phenomenon and revealing its nature contributes to understanding consciousness. The ecological momentary assessment (EMA), in which subjects report a momentary mental state, is a suitable method to investigate MW in a natural environment. Previous studies employed EMA to study MW and attempted to answer the most fundamental question: How often do we let our minds wander? However, reported MW occupancies vary widely among studies. Further, while some experimental settings may induce bias in MW reports, these designs have not been explored. Therefore, we searched PubMed and Web of Science for articles published until the end of 2020 and systematically reviewed 25 articles, and performed meta-analyses on 17 of them. Our meta-analysis found that people spend 34.504% of daily life in mind-wandering, and meta-regression revealed that using subject smartphones for EMA, frequent sampling, and long experimental duration significantly affect MW reports. This result indicates that EMA using subject smartphones may tend to collect sampling under habitual smartphone use. Furthermore, these results indicate the existence of reactivity, even in MW research. We provide fundamental knowledge of MW and discuss rough standards for EMA settings in future MW studies.

## Introduction

Today, ecological momentary assessment (EMA) is an indispensable method to investigate the nature of mind-wandering (MW^1^). MW, a phenomenon in which one’s attention drifts away from the current task or situation, has received attention from psychologists and cognitive neuroscientists. People spend much of the time allowing their minds to wander^2^, and during MW, they incubate ideas, which contributes to creativity^2,3^, MW is a worthy theme of cognitive psychology and neuroscience. Furthermore, clinical psychologists also focus on MW because it may worsen mood and cause depression^4–6^. It is thought to be related to a powerful psychotherapy mechanism called “mindfulness-based intervention”^7,8^. While some behavioral^9,10^, neural^11,12^, and other physiological indices^11,13,14^ have been suggested as indirect approaches, the only direct assessment tool for private MW experience is self-reporting^15^. It is widely thought that EMA should be used when assessing MW in daily life, because it is a self-reporting method that can capture MW that is progressing unconsciously. In EMA, questions are delivered to participants using smartphones or similar devices while they are spending their time normally, and participants respond to these inquiries about their current mental states^16^. While MW often progresses unconsciously, abruptly delivered probes make participants aware of their MW and enable researchers to observe unconsciously progressing MW. Moreover, while retrospective reports of MW have the risk of recall bias^17^, EMA asking about momentary states can avoid such risk^15,18^.

Indeed, many previous studies employed EMA to investigate MW and reported major findings, including the amount of time spent in MW during normal activities. Though EMA is the best way to address this issue, reported MW rates vary widely between articles. We hypothesized that differences in two aspects of study design may explain this inconsistency. First, we hypothesized that differences in the number of questions asked between scenarios when MW exists and when it does not, reduce MW reporting. In some MW EMA studies, contents of sampling were branched, based on the answer. In a typical design with branching questions, the first question asks whether participants are absorbed in MW. If they answer “Yes”, then they are required to answer additional questions about characteristics of MW, e.g., emotional value, temporal orientation, etc. However, if they answer “No”, additional questions are skipped and the sampling ends immediately or soon thereafter. While such branching eliminates meaningless questions and allows subjects to save time, it may encourage subjects to answer “No” in order to avoid answering so many questions. Second, we hypothesized that utilizing participant smartphones biases the reported MW rate, because sampling may concentrate on the smartphone-use context. Some MW EMA studies lent subjects an electronic device designed for EMA and probes were submitted to it, and others sent probes through subject smartphones using an app for EMA or messages. When smartphones are employed, subjects are sometimes unaware of probe notifications, but this is not possible if the probe arrives while participants are actively using their smartphones. Hence, EMA may be prone to gathering answers when participants are using their smartphones. Because the phones provide interesting content for users, people may experience less MW while using them. Hence, this overrepresentation of smartphone-use may underestimate the MW rate.

Therefore, in this study, we systematically reviewed designs of MW EMA studies and meta-analyzed them. First, we statistically synthesized reported MW rates and calculated the average MW occupancy. Then, we performed meta-regression analyses and investigated experimental settings that altered MW reports. As mentioned above, we hypothesized that question branching and smartphone use affect reported MW. Moreover, though this was not originally planned, we explored other experimental parameters having a risk of bias. Finally, based on results of meta-regression and systematic review, we discussed and suggested a rough standard of experimental design when using EMA for MW research.

Even though there are articles reviewing EMA studies and summarizing their designs^19–23^ and other studies empirically exploring experimental parameters^24–26^, this study, which targeted MW studies regarding healthy people, has clear originality. As EMA designs can be tailored for individual study purposes^20^, different trends regarding experimental parameters can be seen in MW EMA studies. Moreover, most importantly, this is the first study to investigate the risk of bias due to specific EMA design settings in MW reports.

## Methods

### Eligibility criteria

All articles published in English through 2020, and fulfilling the following criteria were included in this systematic review: (1) reporting studies conducting an EMA and (2) EMAs targeting MW, including task-unrelated thought, self-generated thought, spontaneous thought, stimulus-independent thought, and day-dreaming.

From these, articles matching any of the following exclusion criteria were excluded from the meta-analysis: (1) The mean and *SD* of MW rate were not provided by authors, and the reviewer could not calculate them from the report or the available data; (2) the MW rate was reported only from an atypical subject group, e.g., those with specific disorders or with special training; or (3) subjects who reported few or excessive MW were rejected and MW rate before rejection was not provided.

To check the publication date, we referred only to the date on which the issue of the journal was published (coded as *PD* in PubMed) and not other types of publication dates, such as the date of electronic publication.

### Reviewing process

We searched PubMed and Web of Science on 21 July 2021 (and repeated the search on 8 February 2022) for articles using the following terms: '(mind-wandering OR "mind wandering" OR "task-unrelated thought" OR "task unrelated thought" OR "self-generated thought" OR "spontaneous thought" OR "day-dreaming" OR "stimulus-independent thought" OR "stimulus independent thought") AND ("experience sampling" OR "ecological momentary assessment" OR "ambulatory assessment")'.

Reviewers independently checked abstracts and examined eligibility for systematic reviews and meta-analyses in duplicate. Articles judged ineligible by both reviewers were excluded. We retrieved the full text of articles that passed the first triage. Two reviewers independently reviewed full texts and extracted data items. Their evaluations were compared and conflicts were resolved by discussions.

We extracted the duration of the experiment, sampling frequency, counts of question items per probe, rewards, characteristics of subjects, numbers of analyzed and removed subjects, criteria for subject removal, EMA tool, mean compliance rate, and mean MW rate. The MW rate is the total number of MW answers per subject divided by the total number of probes per subject, and the mean MW rate is its average. When the sampling frequency varied by subject or by experiment day, or when the question items in one probe varied because of branching, we calculated their expected value. For example, in McVay et al. (2009)^27^, a probe contained 24 items if MW existed and 19 if it did not, and the mean MW rate was 30%. In this article, we calculated the expected value as (24 × 0.3) + (19 × 0.7) = 20.5. If the study examined MW using a Likert scale, the central point or higher was treated as MW^28^. When these data items could not be extracted, we acquired the raw data and calculated them if the data were publicly available. Otherwise, we asked the investigators about unclear items by email. We excluded articles with unclear or missing items from the analysis, if efforts to clarify or recover them were unsuccessful.

Though we had planned during the pre-registration to summarize the time limit for responses and instructions for subjects on what to do when they could not answer immediately, we abandoned these objectives because few articles reported them. Additionally, we did not list all question items because they were more numerous than expected.

### Data synthesis

We investigated homogeneity between studies with *Q* and *I*^2^ and conducted a random-model, meta-analysis using the DerSimonian-Laird method. The primary outcome was the MW rate, that is, the ratio of affirmative answers regarding engaging in MW. The reported mean MW rates and their confidence intervals were displayed in a forest plot. To determine the average, we performed meta-regressions and investigated the relation between MW rate and some variables. The main independent variables were differences in the number of questions between “if MW exists” and “if it does not”, and the EMA tool, i.e., whether subject smartphones were used for EMA, and both were study-level variables. Though the first independent variable was not part of the original design, we decided to investigate it during the review of articles.

Along with the originally planned hypothesis testing, we performed exploratory analyses. First, we conducted additional meta-regression and explored other factors that increase or decrease MW answers. We used the duration, sampling frequency, and the number of questions per probe. *p* values for estimated slopes were corrected using the Benjamini–Hochberg procedure. Second, we repeated the same analyses on the reported compliance rate to help researchers decide on parameters for avoiding bias in MW reports. That is, we summarized the compliance rate in a table (Supplementary Table S1) and a forest plot, conducted meta-regressions, and explored whether the foregoing experimental parameters affect compliance.

We assumed that the risk of publication bias was quite small, because the extracted data, e.g., the MW rate, was not thought to affect publication. Similarly, because the extracted data were fundamental EMA data, they are not likely to be selectively reported. Hence, we did not assess meta-bias. We did not assess biases in individual studies because this review does not target intervention effects, and there are no suggestions regarding possible risk of bias for extracted data.

For statistical synthesis, we used R version 4.1.0 (2021-05-18) and the metafor 3.0.2 package. This study was conducted based on the Preferred Reporting Items for Systematic Reviews and Meta-Analysis (PRISMA, 2020^29^) guidelines, and our protocols were pre-registered on OSF (https://osf.io/m8qk3/?view_only=e2e0a9be1f1f47328635df2331989d7a).

### Additional analyses

After completing the above-mentioned review and data synthesis, we reviewed the articles again and extracted some additional items. We extracted the type of scale asking about MW and the reported ages of participants. Further, we extracted the definition of MW that was given to participants. In other words, we summarized what rate we extracted as the “MW rate.” We extracted this information from questions presented to participants in EMA. However, if authors stated that they introduced the definition of MW to the participants, we ignored the question. Instead, we extracted the definition from the introduction, or if it was unavailable, we extracted the definition described in the paper.

Afterward, we found that almost all articles defined MW as task-unrelated thought (TUT) or stimulus-independent (SI) TUT. Therefore, during supplementary analyses, we conducted the same data synthesis of MW rate with only those articles, from which we extracted TUT or SI TUT as MW.

In the meta-regression of duration and sampling frequency, we found two articles (one in each analysis) that appeared to be outliers. Therefore, as a sensitivity analysis, we repeated these analyses excluding the article that appeared to be an outlier.

## Results

### Study selection

**Figure 1:**
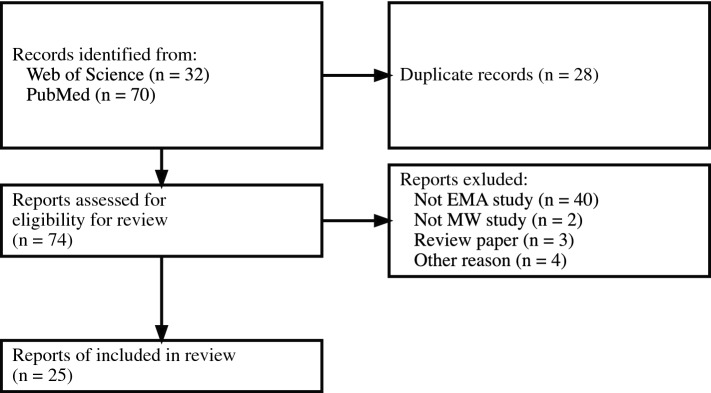
Flow chart of the study selection protocol.

Our flow of study selection is shown in Fig. 1. We found 32 articles in the Web of Science and 70 in PubMed. Of these, 28 were duplicates and were excluded. We also excluded 40 articles for not performing EMA (laboratory experiments only), two that did not target MW, and three that were review papers. Furthermore, a paper^13^ that shared data with an included article^30^, an abstract for a conference^31^, a paper targeting only a clinical sample^32^, and a corrigendum^33^ were rejected (“Other reasons” in Fig. 1). Finally, we included 25 articles for the systematic review.

### Syntheses of experimental parameters

**Figure 2:**
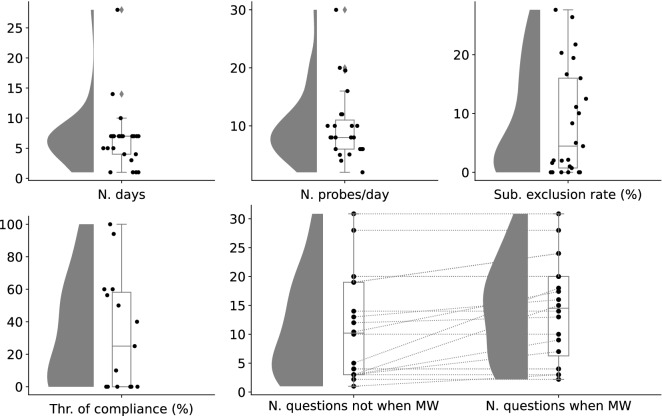
The distribution of experimental parameters of reviewed articles. *N. days* Number of days (Experiment duration), *N. probes/day* Expected number of probes per day, *Sub. exclusion rate (%)* Rate of excluded subjects to recruited subjects, *Thr. of compliance (%)* Threshold of compliance rate (only if authors used compliance rate as a criterion of subject exclusion), *N. questions not when MW* Expected number of questions if MW does not exist, *N. questions when MW* Expected number of questions if MW exists.

We summarize experimental duration (number of days), number of probes per day, expected number of questions in a probe, number of questions if MW did not occur and if it did, reward for subjects, number of included and excluded subjects in EMA, exclusion criteria, whether subject smartphones were utilized, and the mean and *SD* of compliance and MW rates (Supplementary Table S1). Distribution of the number of days and probes per day, subject exclusion rate, the threshold of compliance rate for exclusion, and the number of questions if MW occurred and if it did not are plotted in Fig. 2. The number of included studies, mean, mode in the kernel density estimation, *SD*, and minimum and maximum values of these parameters are provided in Table 1. Of all studies, 45.833% used subject smartphones for EMA. From these, we extracted task-unrelated thought from 16 articles, stimulus-independent task-unrelated thought from 4, stimulus-independent thought without rumination and anxiety from 3, freely-moving task-unrelated thought from 1, and freely-moving mind from 1.

**Table 1:** Summary of experimental parameters.

	N. of articles	Mean	Mode (KDE)	SD	Min	Max
N. days	25	6.640	7.000	5.345	1.000	28.000
N. probes/day	23	9.983	8.000	6.283	2.000	30.000
Sub. exclusion rate (%)	25	8.362	1.980	9.174	0.000	27.632
Thr. of compliance (%)	15	33.026	10.000	35.540	0.000	100.000
N. questions not when MW	20	11.223	5.000	9.048	1.000	30.864
N. questions when MW	20	14.123	16.000	8.758	2.188	30.864

*N. days* Number of days (Experiment duration), *N. probes/day* Expected number of probes per day, *Sub. exclusion rate (%)* Rate of excluded subjects to recruited subjects, *Thr. of compliance (%)* Threshold of compliance rate (only if authors used compliance rate as a criterion of subject exclusion), *N. questions not when MW* Expected number of questions if MW does not exist, *N. questions when MW* Expected number of questions if MW exists.

### Meta-analyses

**Figure 3:**
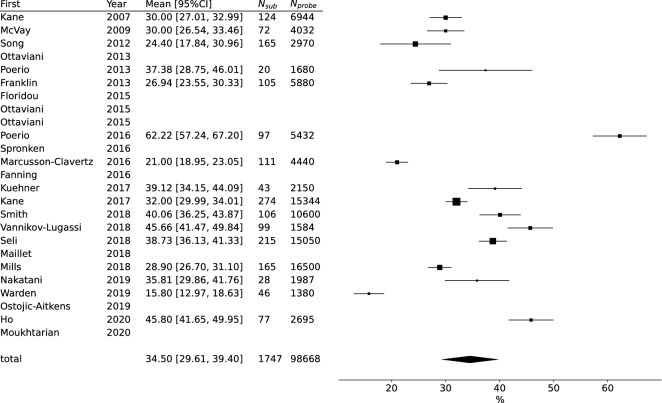
Forest plot of mind-wandering rate. The effect size and corresponding bar were blank if the mean MW rate was unavailable. *First* First author of studies, *Year* Year of publication, *Mean [95%CI]*, Reported mean MW rate and 95% confidence intervals, *N*_*sub*_ Included number of subjects, *N*_*probe*_ Included total number of probes.

**Figure 4:**
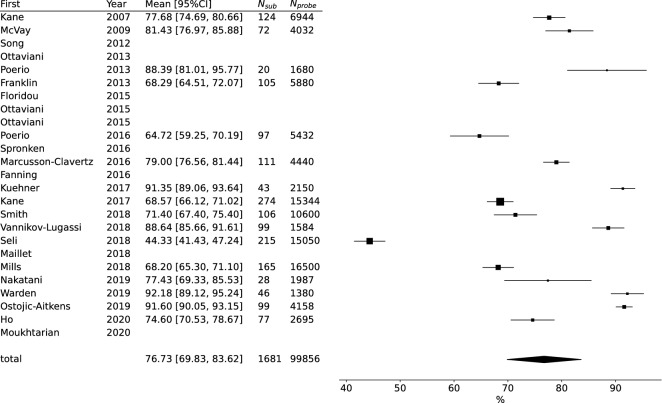
Forest plot of compliance rate. The effect size and corresponding bar are blank if the mean compliance rate was unavailable. *First* First author of studies, *Year* Year of publication, *Mean [95%CI]* Reported mean compliance rate and 95% confidence intervals, *N*_*sub*_ Included number of subjects, *N*_*probe*_ Included total number of probes.

Sixteen articles reported the mean MW rate and 16 reported the mean compliance rate, and we used them to conduct meta-analyses. We found large heterogeneity between reviewed articles (MW rate: *Q* = 534.686, *p* < 0.001, *I*^2^ = 97.195 (95.851–99.075), *N* = 16; compliance: *Q* = 1226.747, *p* < 0.001, *I*^2^ = 98.777 (97.254–99.378), *N* = 16). The meta-analysis indicated that the mean MW rate was 34.504% (*CI* = 29.607–39.401%; Fig. 3) and the mean compliance rate was 76.727% (*CI* = 69.828–83.625%; Fig. 4). The meta-regression showed that utilizing subject smartphones (*Estimate* = 10.56; *p* = 0.03; 95% *CI* = 1.29–19.82), experiment duration (*Estimate* = 1.26; *p* < 0.001; 95% *CI* = 0.6–1.92), and number of probes per day (*Estimate* = − 0.86; *p* = 0.01; 95% *CI* = − 1.51–− 0.2) significantly affected reported MW rates.

**Figure 5:**
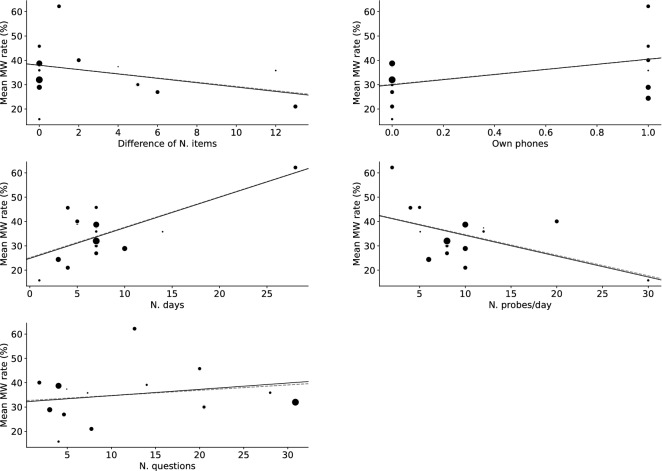
Scatter and meta-regression plot of mind-wandering rate. Marker sizes reflect article sample sizes. The solid line denotes the meta-regression line biased due to article weights. The dashed line denotes the unweighted regression line. *N. days* Number of days (Experiment duration), *N. probes/day* Expected number of probes per day, *Sub. exclusion rate (%)* Rate of excluded subjects to recruited subjects, *Thr. of compliance (%)* Threshold of compliance rate (only if authors used compliance rate as a criterion of subject exclusion), *N. questions not when MW* Expected number of questions if MW does not exist. *N. questions when MW* Expected number of questions if MW exists.

**Figure 6:**
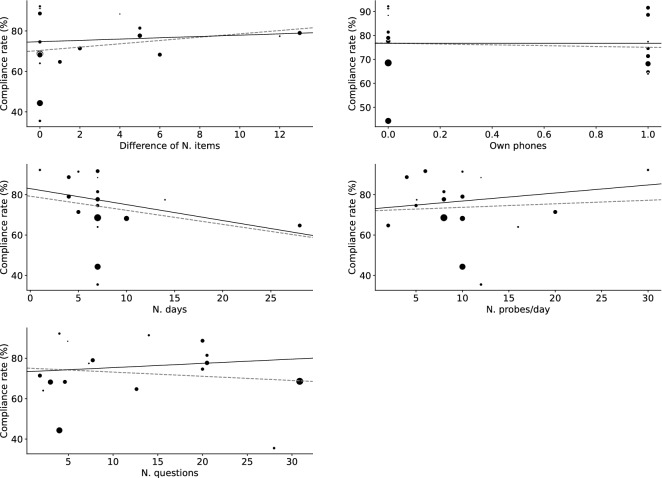
Scatter and meta-regression plot of compliance rate. Marker sizes reflect article sample sizes. The solid line is the meta-regression line biased by article weights. The dashed line denotes the unweighted regression line. *N. days* Number of days (Experiment duration), *N. probes/day* Expected number of probes per day, *Sub. exclusion rate (%)* Rate of excluded subjects to recruited subjects, *Thr. of compliance (%) *Threshold of compliance rate (only if authors used compliance rate as a criterion of subject exclusion), *N. questions not when MW* Expected number of questions if MW does not exist, *N. questions when MW* Expected number of questions if MW exists.

Results of meta-regression for MW rate are shown in Table 2 and Fig. 5, and for compliance, in Table 3 and Fig. 6. The regulating definition of MW (only TUT or SI TUT articles were included) did not cause different results (Supplementary Results and Supplementary Table S2). The meta-regression of duration and sampling frequency without the outlier article did not show significant effects on MW rates (Supplementary Results and Figure).

**Table 2:** Meta-regression results regarding MW rate.

	Estimate	*SE*	*Z*	*p*	*p*_*adj*_	low	high	N
Mean MW rate (%)								
*Difference of N. items*								
Intercept	37.984	3.153	12.047	0.000		31.804	44.164	15
*Beta*	− 0.899	0.597	− 1.507	0.132	0.165	− 2.069	0.270	
*N. days*								
Intercept	24.830	3.234	7.679	0.000		18.493	31.168	16
*Beta*	1.259	0.335	3.756	0.000	0.001	0.602	1.916	
*N. probes/day*								
Intercept	42.883	3.971	10.798	0.000		35.099	50.666	16
*Beta*	− 0.857	0.336	− 2.552	0.011	0.027	− 1.515	− 0.199	
*N. questions*								
Intercept	32.090	4.616	6.952	0.000		23.043	41.137	15
*Beta*	0.260	0.317	0.820	0.412	0.412	− 0.361	0.880	
*Own phones*								
Intercept	29.917	3.110	9.620	0.000		23.822	36.012	16
*Beta*	10.555	4.726	2.234	0.026	0.043	1.294	19.817	

*Difference of N. items* Differences in the number of questions asked between scenarios when MW exists and when it does not, *N. days* Number of days (Experiment duration), *N. probes/day* Expected number of probes per day, *N. questions* Expected number of questions.

**Table 3:** Meta-regression results regarding compliance rate.

	Estimate	*SE*	*Z*	*p*	*p*_*adj*_	low	high	N
Compliance rate (%)								
*Difference of N. items*								
Intercept	74.694	4.733	15.780	0.000		65.416	83.971	15
*Beta*	0.323	0.901	0.359	0.720	0.899	-1.442	2.088	
*N. days*								
Intercept	82.939	5.706	14.536	0.000		71.756	94.123	16
*Beta*	− 0.789	0.583	− 1.353	0.176	0.881	− 1.932	0.354	
*N. probes/day*								
Intercept	72.851	6.563	11.100	0.000		59.988	85.713	16
*Beta*	0.397	0.558	0.711	0.477	0.899	− 0.697	1.490	
*N. questions*								
Intercept	73.244	6.335	11.562	0.000		60.828	85.660	15
*Beta*	0.210	0.435	0.482	0.630	0.899	− 0.644	1.064	
*Own phones*								
Intercept	76.733	4.810	15.954	0.000		67.307	86.160	16
*Beta*	− 0.014	7.287	− 0.002	0.998	0.998	− 14.297	14.268	

*Difference of N. items*, Differences in the number of questions asked between scenarios when MW exists and when it does not. *N. days* Number of days (Experiment duration), *N. probes/day* Expected number of probes per day, *N. questions* Expected number of questions.

## Discussion

This study systematically collected articles, reviewed them to summarize experimental parameters, calculated the mean proportion of MW, and investigated variables that affect the MW rate.

### The definition of MW

A large number of reviewed articles provided the ratio of TUT or SI TUT. Even if an article mainly targeted more narrowly defined MW, e.g., TUT without meta-awareness, in many cases, the first question was about TUT and its details in subsequent questions. TUT was defined as thoughts or images not directed toward one’s current activity^34^, and SI means that it comes from the internal mental process and is not related to the immediate environment^35^. Even if articles that adopted different definitions of MW were rejected from data synthesis, the statistical significance did not differ. Note that this does not imply that the difference in MW type did not affect MW report, since we just tested the result without articles treating freely-moving thought and did not compare MW type thoroughly.

### Meta-analysis

While a meta-analysis showed that people engage in MW for one-third of their waking hours, large heterogeneity between articles existed. Not only the hypothesized variables, but also other experimental parameters affected the MW report. The more frequently subjects received probes or the shorter the duration of the experiment, the less they reported MW. These results may indicate that reactivity (observer effect; Hawthorne effect) occurs even in MW EMA research to some extent. Reactivity refers to the effect on research outcomes derived from the study protocol^36^. Receiving constant EMA probes (or expectation of this) could lead subjects be more aware of their MW, and their MW may be shortened. The assumption that reactivity occurred explains the effects of experimental duration and sampling frequency simultaneously. More frequent sampling is thought to reinforce reactivity^37^; thus, the number of probes per day decreased MW reports in our meta-analysis. Considering that this reactivity ceased as participants became accustomed to it^38^, it is reasonable that longer experiments increased reported MW.

From the scatter plot (Figs. 5 and 6), both regressions included one article that appeared to be an outlier^39,40^. In meta-analyses, outlier rejection is not recommended^41^. Particularly, rejection of such articles should be carefully conducted. While outliers in measured data indicate potential measurement errors and are usually rejected, in the current case, authors intentionally designed settings are outliers rather than measured data. However, in any case, sensitivity analyses without these articles did not show the same significance and we cannot argue that those were robust results.

Using meta-regression, we found that utilizing subject smartphones for EMA significantly increased the MW rate, contrary to our hypothesis that since smartphones provide users with interesting content, MW would be reduced. We interpret this result to mean that people experience more MW while using smartphones. Previous research indicated that 60% of smartphone use was habitual and absent-minded^42^. It has been strongly suspected that people report MW during habitual smartphone use, and indeed the propensity for habitual use correlates well with the MW trait^43^. In smartphone-based EMA, reports during habitual use may be overrepresented.

Otherwise, given the potential reactivity found in the current meta-analysis, this smartphone effect can also be explained as reactivity. Compared to habitually carried smartphones, additional EMA devices may more readily remind subjects of the measurement. Hence, extra wearable devices to gather additional data, e.g., heart rate, can also enhance reactivity (no articles used subject smartphones or extra devices in our meta-analysis). However, the current systematic review and meta-analysis cannot reveal which interpretation (overrepresentation of smartphone using condition vs. enhancement of reactivity) is correct. Future studies that directly compare EMA protocols with and without subject smartphones should reveal it.

However, we did not find a significant effect from use of branching questions in cases in which participants reported MW. This result did not support the contention that the risk of allowing subjects to skip questions leads to bias in the report. However, it is still possible that branching questions selectively bias MW reports in unmotivated participants, e.g., people participating in an experiment only for cash reward.

### Suggestions regarding experimental parameters

Based on the aforementioned meta-analysis and a summary of previous studies, some suggestions about experimental parameters in future MW EMA studies are appropriate. The meta-analysis showed that using subject smartphones affects MW reports; however, it did not support the supposition that using a smartphone or a dedicated device (or both) leads to bias. If using subject smartphones drives bias, it would be effective to include a question asking about momentary smartphone use or collecting use data, so as to consider whether subjects used them when they received probes. To cope with the potential effect of EMA-dedicated devices or extra wearable devices, we can use a trial period to acclimate the user to the device. Future studies are expected to reveal which (or both) approaches are effective for more precise EMA.

The mode value of experimental duration varied, centering on a week, except for a paper performing a 28-day experiment^39^. Given that reactivity indicated by our meta-analysis and previous studies showed that longer experiments did not degrade compliance^21–23^, we recommend a long-term experiment if an assessment in a maximally natural environment is needed. However, from this study, we cannot propose a duration for which the observer effect would cease. At present, we suggest a seven-day experiment for compatibility with previous studies. Notably, when a long-term experiment is performed, researchers need to pay attention to the possibility that the earlier and later parts of individual datasets may not be homogeneous.

Sampling frequency also appears to produce reactivity. Considering that the mode value of frequency in previous research was 8.0/day, and a study reported that the quality and compliance rate were unchanged by fewer than 9 probes/day in previous studies^24,25^, we suggest using the minimum required frequency, which ideally, is fewer than 9.

While the numbers of questions varied, all except for one study (33 maximum^44^) adopted fewer than 30 items. We think that future studies should set the number of questions equal to the number of aspects of MW being investigated; however, to maintain compatibility with previous studies, answer quality^24,25^, and compliance^24^, we recommend restricting it to under 30 questions. Though there was no evidence indicating a risk caused by branching questions, further studies are needed. If we want to avoid branching, we can allow subjects to answer based on the latest MW experience if their minds are not wandering when the probe arrives^39^.

When designing an experiment using EMA, it is also beneficial to consider this recommendation driven by lab-based experiments. In the probe-caught paradigm, participants sometimes receive and answer a probe asking about their momentary attentional state while performing a cognitive task demanding relatively simple focusing. We may be able to understand EMA as a probe-caught paradigm extended from the laboratory to daily life (aside from the history of method development). Therefore, it would be beneficial to consider knowledge and recommendations from lab-based probe-caught paradigm studies. One article^15^ reviewed probe-caught paradigm experiments and suggested some recommendations about probe framing and response options, etc. Another study^17^ experimentally investigated the effect of different probe types and recommended that probes ask about the content of thought, rather than the intensity of MW. These proposals would be compatible with EMA studies.

### Limitations and Future Directions

While we tried to comprehensively collect articles with pre-registered search strategies, it is possible that we missed some papers. Particularly, we might have missed several articles published before EMA-related terms such as “EMA” or “experience sampling” became widespread among scientists. However, we think we achieved the study aim, which was to contribute to future EMA MW research, by covering relatively recent papers.

Readers should note that our review and meta-analysis did not include Killingsworth and Gilbert (2010)^45^, the largest EMA MW study conducted, because EMA-relating words were not included. This study includes an enormous number of participants (2,250) so including this study would have heavily biased the results. The design of that study deviated strongly from other studies in that people freely download the app and started and terminated their participation in the study. The enormous contribution of this study notwithstanding, its unmanaged design would have biased our results in ways that would be difficult to control, so, we felt that removing it from the present meta-analysis was prudent.

Since the number of MW EMA studies is still growing, re-performing the meta-analysis of EMA MW articles after decade or more is expected not only to validate our current results, but also to resolve some limitations of the current study. First, such a future study would summarize differences in the occupancy of each MW type. Investigating the effect of MW types is beyond the intent of the current research and it is difficult to determine it from the collected articles because they mainly targeted TUT. However, this is a crucial topic for MW studies, given that TUT has different associations with mental problems^46^ and brain activity^47^ depending on whether it is stimulus-independent or stimulus-dependent, and future meta-analysis is expected to evaluate it. Moreover, with more articles, it will be possible to statistically control some characteristics of subjects in meta-analyses. For example, since the age of subjects correlates with an MW trait^48^, it can be entered as a co-variate.

## Supplementary Information

Supplementary Information 1.

Supplementary Information 2.

## Data Availability

Data and codes that support the findings of this study are available in the supplementary information.
