# Supplementary material for: Ecological momentary assessment of mind-wandering: meta-analysis and systematic review
Source: Sci Rep. 2023 Feb 18;13:2873. doi: 10.1038/s41598-023-29854-9 (PMC9938857; doi:10.1038/s41598-023-29854-9)
Supplement: Supplementary file 2 — Supplementary Information 2. [file 41598_2023_29854_MOESM2_ESM.pdf]

Ecological Momentary Assessment of Mind-Wandering: Meta-Analysis and Systematic Review (Supplementary Material)

Issaku Kawashima[1*], Tomoko Hinuma[1], and Saori C. Tanaka[1,2*]

Supplementary Results

From 16 articles reporting the mean MW rate, we excluded two studies[1,2] because those papers defined MW as "freely moving thought." We conducted a meta-analysis and meta-regression on the MW rate. The heterogeneity was still large (MW rate: $Q = 509.418$, $p < .001$, $I^2 = 97.448$ (95.877–99.182), $N = 14$). The mean MW rate was 34.535% (CI = 28.859–40.212%).

While the experimental duration and the number of probes per day correlated significantly with the mind-wandering rate, some articles might serve as outliers. Therefore, for sensitivity analyses, we removed the outlying article and performed meta-regression analyses. Meta-regression without an article[3] did not show a significant correlation between the number of days and the mind-wandering rate (*Estimate* = 0.9; $p = .22$; 95% CI = -0.53—2.33 Moreover, meta-regression without an article[4] did not show a significant correlation between the number of probes per day and mind-wandering rate (*Estimate* = -0.73; $p = .21$; 95% CI = -1.88—0.41).

Supplementary Tables

Supplementary Table S1. List of reviewed articles and extracted items.

	N. days	N. probes/day	N. questions	Definition of MW	Reward	Characteristics of subjects	Age	Final N. sub.	Removed N. sub.	Exclusion criteria	Own phones	Compliance (%)	Mean MW (%)	Scale	Note
Kane et al., 2007[5]	7	8	20.50 (19 – 24)	TUT	cr. or USD100	Undergraduates	19.34 ± 2.41 (17–35)	124	2		N	77.68 (16.96)	30.00 (17.00)	Dichotomous	
McVay et al., 2009[6]	7	8	20.50 (19 – 24)	TUT	gift-card (lottery)	Undergraduates	18–35	72	0	-	N	81.43 (19.29)	30.00 (15.00)	Dichotomous	
Song et al., 2012[7]	3	6		SI TUT	CNY30	Undergraduates without history of mental disorder	20.05 ± 1.53 (18–29)	165	0	-	Y		24.40 (43.00)	Dichotomous	
Ottaviani et al., 2013[8]	1			SI TUT excluding worry and rumination	EUR25	Students at Sapienza University of Rome	24.50 ± 4.9	40	5	1 dropouts; 3 exessive artifacts* or consistent error	N			Dichotomous	*In physiological measurements

	N. days	N. probes/day	N. questions	Definition of MW	Reward	Characteristics of subjects	Age	Final N. sub.	Removed N. sub.	Exclusion criteria	Own phones	Compliance (%)	Mean MW (%)	Scale	Note
Poerio et al., 2013[3]	7	12*	4.93 (3 – 7)$^{+}$	TUT		14 were postgraduates, 9 were in full-time employment and one was unemployed	24.17 ± 2.9	20	4	-	N	88.39 (16.84)$^{+}$	37.38 (19.69)$^{+}$	Dichotomous	*6 pairs
Franklin et al., 2013[9]	7	8	4.62 (3 – 9)	TUT	USD30, and USD50 (lottery)	People recruited using posted flyers in UBC campus	23.1 ± 7.4	105	0	-	N	68.29 (19.76)$^{+}$	26.94 (17.74)$^{+}$	Dichotomous	
Floridou et al., 2015[10]	7	6		SI TUT		People registered in Goldsmiths Earwormery Questionnaire database	18–72	38	2	Low compliance rate (threshold is 100%)	Y			Dichotomous	
Ottaviani et al., 2015[11]	1	19.548	(13 – 16)	SI TUT excluding worry and rumination	EUR25	University students and employees	26.68	42	8	3 dropouts; 5 excessive artifacts^{*1} or consistent error	N			Dichotomous	^{*1}In physiological measurements; ^{*2} It is calculated collapsing “mind-wandering” and “perseverati

	N. days	N. probes/day	N. questions	Definition of MW	Reward	Characteristics of subjects	Age	Final N. sub.	Removed N. sub.	Exclusion criteria	Own phones	Compliance (%)	Mean MW (%)	Scale	Note
															ve cognition”
Ottaviani et al., 2015[12]	1		10 (10 – 10)	TUT excluding worry and rumination	USD35	People who met diagnostic criteria for a current major depressive episode and healthy controls	30.10 ± 10.50	36	0		N			Free description	Only the data from healthy group was used in our study
Poerio et al., 2016[13]	28	2	12.62 (12 – 13)	SI TUT	cr. and GBP10		19.34 ± 2.34	97	2	Dropout	Y	64.72 (27.47)$^{+}$	62.22 (25.00)$^{+}$	Dichotomous	
Spronken et al., 2016[14]	7	6	12.11 (10.41 – 17.41)	TUT	EUR10	People recruited in the city, local soccer club (n=35), and local hockey club (n=19), zen organization (n=40)	39.6 ± 14.1 (18–72)	162	45	Low compliance rate (threshold is 50%)	Y			Dichotomous	
Marcusson-Clavertz et al., 2016[15]	4	10	7.73 (5 – 18)	SI TUT	Two cinema tickets	Most participants were	24.75 ± 4.62(18–41)	111	0	-	N	79.00 (13.12)	21.00 (11.00)	Dichotomous	

	N. days	N. probes/day	N. questions	Definition of MW	Reward	Characteristics of subjects	Age	Final N. sub.	Removed N. sub.	Exclusion criteria	Own phones	Compliance (%)	Mean MW (%)	Scale	Note
						undergraduate students									
Fanning et al., 2016[16]	7	16	2.19 (2.19 – 2.19)	TUT		People in Midwestern university	20.5 ± 1.5 (18–25)	33	3	Low compliance rate (threshold is 25%)	Y	64.00		Dichotomous	
Kuehner et al., 2017[17]	5	10	14 (14 – 14)	TUT	cr. and EUR20	Students of the University of Mannheim	21.74 ± 3.14 (19–32)	43	2	Excessive missing data	N	91.35 (7.67)^{o}	39.12 (16.63)^{o}	Likert	
Kane et al., 2017[18]	7	8	30.86 (30.86 – 30.86)$^{+}$	TUT	USD50, and gift-card (lottery)	Undergraduates at the University of North Carolina at Greensboro	18.74 ± 1.79 (18–35)	274	2	Low variance of answer to EMA (56% and 39%)	N	68.57 (20.71)	32.00 (17.00)	Dichotomous	
Smith et al., 2018[1]	5	20	1.82 (1 – 3)	Freely moving mind	cr.	People recruited from a large public Canadian university		106	38	Low compliance rate (threshold is 60%)	Y	71.40 (21.00)	40.06 (20.00)^{o}	Likert	
Vannikov-Lugassi et al., 2018[19]	4[*1]	4	20 (20 – 20)$^{+}$	TUT	ILS150	Undergraduate students	23.96 ± 1.97	99	2	Dropout	Y	88.64 (15.11)$^{+}$	45.66 (21.25)$^{+}$	Likert	[*1]While some participants voluntarily

	N. days	N. probes/day	N. questions	Definition of MW	Reward	Characteristics of subjects	Age	Final N. sub.	Removed N. sub.	Exclusion criteria	Own phones	Compliance (%)	Mean MW (%)	Scale	Note
															continued until 5th day, its data were ignored in this paper; *2The item of (c) in “Thinking about the Present” was treated as MW question
Seli et al., 2018[20]	7	10	4 (4 – 4)	TUT		Undergraduate students		215	24	Low compliance rate (threshold is 10%)	N	44.33 (21.72)º	38.73 (19.46)º	Dichotomous	*Two conditions (dichotomous or multi-level probe) were collapsed in this study
Maillet et al., 2018[21]	7	12	28 (28 – 28)	TUT	USD100	31 young and 20 older adults	40.81 (18–77)	51	13	Low compliance rate (threshold is 94.048%)		35.53	35.90	Dichotomous	Young and older subject groups were merged

	N. days	N. probes/day	N. questions	Definition of MW	Reward	Characteristics of subjects	Age	Final N. sub.	Removed N. sub.	Exclusion criteria	Own phones	Compliance (%)	Mean MW (%)	Scale	Note
Mills et al., 2018[2]	10	10	3 (3 – 3)	Freely moving TUT	cr.	A large public Canadian university		165	63	Low compliance rate (threshold is 60%)	Y	68.20 (19.00)	28.90 (14.40)*	Dichotomous	*From several types of thought they reported, we extracted freely-moving–off-task thought as MW
Nakatani et al., 2019[22]	14	5.071	7.30 (3 – 15)	TUT	cr.	Students in psychology program at KU Leuven	19	28	4	Low compliance rate (threshold is 56.338%)	Y	77.43 (21.86)+	35.81 (16.06)+	Dichotomous	
Warden et al., 2019[4]	1	30	4 (4 – 4)*1	TUT		Young and older groups	49.15 (18–90)	46	1	Consistent error	N	92.18 (10.60)+	15.80 (9.80)+	Free description	Young and older subject groups were merged; We included only the study 2;*1Two free descriptions about thoughts and action,

	N. days	N. probes/day	N. questions	Definition of MW	Reward	Characteristics of subjects	Age	Final N. sub.	Removed N. sub.	Exclusion criteria	Own phones	Compliance (%)	Mean MW (%)	Scale	Note
															and two items[*2]From several types of thought they reported, we extracted spontaneous task-unrelated thought as MW.
Ostojic-Aitkens et al., 2019[23]	7	6		TUT	cr.	University students	20.75 ± 2.83 (18–34)	99	1	Low compliance rate	Y	91.60 (7.89)		Likert	
Ho et al., 2020[24]	7	5	20 (20 – 20)	TUT	cash or cr.	Undergraduate and postgraduate student bodies at the University of York	19.66 ± 1.62 (18–27)	77	0	-	Y	74.60 (18.20)[+]	45.80 (18.60)[+]	Likert	
Moukhtarian et al., 2020[25]	5	8		TUT	Travel expense and GBP50	28 ADHD, 19 BPD, 22 comorbid ADHD+BPD, and 29 control females	27.1 ± 5.2	29	7[*]	Low compliance rate (threshold is 40%)	N			Score	Only the data from healthy group was used in this study;[*]This may include

	N. days	N. probes/day	N. questions	Definition of MW	Reward	Characteristics of subjects	Age	Final N. sub.	Removed N. sub.	Exclusion criteria	Own phones	Compliance (%)	Mean MW (%)	Scale	Note
															subjects in clinical group

[+], values calculated by the authors. *N. days*, Number of days; Experiment duration.

N. probes/day, Expected number of probes per day.

N. questions, Expected number of question items in one probe (number of questions in case of MW does not exist – that of MW exists).

Definition of MW, Definition of mind-wandering we extracted.

TUT, task-unrelated thought.

SI, Stimulus-independent.

Reward, Rewards for subjects.

USD, United States dollar.

cr., Course credit.

CNY, Chinese yuan.

EUR, Euro.

GBP, Pound sterling.

ISL, Israeli new shekel.

Final N. sub., Final number of subjects; Number of participants after rejection.

Removed N. sub., Excluded number of subjects.

Exclusion criteria, Criteria for subject exclusion.

Own phones, whether subjects' own smartphones were used (Y: Yes, N: No).

Compliance (%), Compliance rate (%) and *SD*; the rate wherein they responded to received probes.

Mean MW (%), Mean mind-wandering rate and *SD*; Study-level average of the rate wherein they answered that their mind wandered.

Scale type, Type of scale used to assess mind-wandering.

Dichotomous, Binary question (i.e., Yes or No) or question in which subjects choose one from multiple choices.

Likert, Likert scale.

Score, Scale 0 to 100.

Supplementary Table S2. Meta-regression results of MW rate regulating MW type

	Estimate	*SE*	*Z*	*p*	$p_{adj.}$	low	high	N
Mean MW rate (%)								
Difference of N. items								
Intercept	38.810	3.743	10.369	0.000		31.474	46.146	13
Beta	-1.005	0.662	-1.520	0.129	0.161	-2.302	0.291	
N. days								
Intercept	24.177	3.233	7.477	0.000		17.840	30.514	14
Beta	1.343	0.328	4.098	0.000	0.000	0.701	1.985	
N. probes/day								
Intercept	44.628	4.060	10.993	0.000		36.672	52.585	14
Beta	-1.120	0.365	-3.071	0.002	0.005	-1.834	-0.405	
N. questions								
Intercept	31.203	6.001	5.200	0.000		19.442	42.965	13
Beta	0.309	0.384	0.804	0.421	0.421	-0.444	1.061	
Own phones								
Intercept	29.895	2.929	10.205	0.000		24.153	35.637	14
Beta	13.168	4.962	2.654	0.008	0.013	3.443	22.894	

Supplementary Figure.

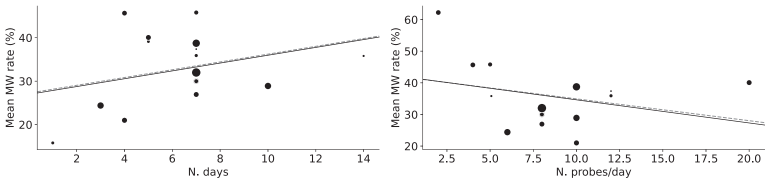

Supplementary Figure. Scatter and meta-regression plot of mind-wandering rate

Marker sizes reflect article sample sizes. The solid line denotes the meta-regression line, biased due to article weights. The dashed line denotes the unweighted regression line.

References for Supplementary Tables

1. Smith, G. K., Mills, C., Paxton, A. & Christoff, K. Mind-wandering rates fluctuate across the day: Evidence from an experience-sampling study. *Cognitive research: principles and implications* **3**, 54 (2018).

2. Mills, C., Raffaelli, Q., Irving, Z. C., Stan, D. & Christoff, K. Is an off-task mind a freely-moving mind? Examining the relationship between different dimensions of thought. *Conscious. Cogn.* **58**, 20–33 (2018).

3. Poerio, G. L., Totterdell, P. & Miles, E. Mind-wandering and negative mood: Does one thing really lead to another? *Conscious. Cogn.* **22**, 1412–21 (2013).

4. Warden, E. A., Plimpton, B. & Kvavilashvili, L. Absence of age effects on spontaneous past and future thinking in daily life. *Psychological research* **83**, 727–746 (2019).

5. Kane, M. J. *et al.* For whom the mind wanders, and when: An experience-sampling study of working memory and executive control in daily life. *Psychol. Sci.* **18**, 614–621 (2007).

6. McVay, J. C., Kane, M. J. & Kwapil, T. R. Tracking the train of thought from the laboratory into everyday life: An experience-sampling study of mind wandering across controlled and ecological contexts. *Psychon. Bull. Rev.* **16**, 857–63 (2009).

7. Song, X. & Wang, X. Mind wandering in Chinese daily lives - an experience sampling study. *PLOS ONE* **7**, e44423 (2012).

8. Ottaviani, C. & Couyoumdjian, A. Pros and cons of a wandering mind: A prospective study. *Front. Psychol.* **4**, 524 (2013).

9. Franklin, M. S. *et al.* The silver lining of a mind in the clouds: Interesting musings are associated with positive mood while mind-wandering. *Front. Psychol.* **4**, 583 (2013).

10. Floridou, G. A. & Müllensiefen, D. Environmental and mental conditions predicting the experience of involuntary musical imagery: An experience sampling method study. *Conscious. Cogn.* **33**, 472–86 (2015).

11. Ottaviani, C., Medea, B., Lonigro, A., Tarvainen, M. & Couyoumdjian, A. Cognitive rigidity is mirrored by autonomic inflexibility in daily life perseverative cognition. *BIOLOGICAL PSYCHOLOGY* **107**, 24–30 (2015).

12. Ottaviani, C. *et al.* Cognitive, behavioral, and autonomic correlates of mind wandering and perseverative cognition in major depression. *FRONTIERS IN NEUROSCIENCE* **8**, (2015).

13. Poerio, G. L., Totterdell, P., Emerson, L.-M. & Miles, E. Social daydreaming and adjustment: An experience-sampling study of socio-emotional adaptation during a life transition. *Front. Psychol.* **7**, 13 (2016).

14. Spronken, M., Holland, R. W., Figner, B. & Dijksterhuis, A. Temporal focus, temporal distance, and mind-wandering valence: Results from an experience sampling and an experimental study. *Conscious. Cogn.* **41**, 104–18 (2016).

15. Marcusson-Clavertz, D., Cardena, E. & Terhune, D. B. Daydreaming style moderates the relation between working memory and mind wandering: Integrating two hypotheses. *JOURNAL OF EXPERIMENTAL PSYCHOLOGY-LEARNING MEMORY AND COGNITION* **42**, 451–464 (2016).

16. Fanning, J. *et al.* Physical activity, mind wandering, affect, and sleep: An ecological momentary assessment. *JMIR MHEALTH AND UHEALTH* **4**, (2016).

17. Kuehner, C., Welz, A., Reinhard, I. & Alpers, G. W. Lab meets real life: A laboratory assessment of spontaneous thought and its ecological validity. *PLOS ONE* **12**, (2017).

18. Kane, M. J. *et al.* For whom the mind wanders, and when, varies across laboratory and daily-life settings. *Psychol. Sci.* **28**, 1271–1289 (2017).

19. Vannikov-Lugassi, M. & Soffer-Dudek, N. No time like the present: Thinking about the past and the future is related to state dissociation among individuals with high levels of psychopathological symptoms. *Front. Psychol.* **9**, 2465 (2018).

20. Seli, P. *et al.* How pervasive is mind wandering, really? *Conscious. Cogn.* **66**, 74–78 (2018).

21. Maillet, D. *et al.* Age-related differences in mind-wandering in daily life. *Psychology and aging* **33**, 643–653 (2018).

22. Nakatani, C., Ganschow, B. & Leeuwen, C. van. Long-term dynamics of mind wandering: Ultradian rhythms in thought generation. *Neuroscience of consciousness* **2019**, niz007 (2019).

23. Ostojic-Aitkens, D., Brooker, B. & Miller, C. J. Using ecological momentary assessments to evaluate extant measures of mind wandering. *Psychological assessment* **31**, 817–827 (2019).

24. Ho, N. S. P. *et al.* Facing up to the wandering mind: Patterns of off-task laboratory thought are associated with stronger neural recruitment of right fusiform cortex while processing facial stimuli. *NeuroImage* **214**, 116765 (2020).

25. Moukhtarian, T. R. *et al.* Wandering minds in attention-deficit/hyperactivity disorder and borderline personality disorder. *EUROPEAN NEUROPSYCHOPHARMACOLOGY* **38**, 98–109 (2020).
